# Modulating fear extinction memory by manipulating SK potassium channels in the infralimbic cortex

**DOI:** 10.3389/fnbeh.2014.00096

**Published:** 2014-03-24

**Authors:** Marangelie Criado-Marrero, Edwin Santini, James T. Porter

**Affiliations:** ^1^Department of Physiology and Pharmacology, Ponce School of Medicine and Health SciencesPonce, Puerto Rico; ^2^College of Pharmacy, Nova Southeastern UniversityPonce, Puerto Rico

**Keywords:** amygdala, medial prefrontal cortex, intrinsic excitability, fear conditioning, PTSD, apamin

## Abstract

Fear extinction correlates with increased infralimbic (IL) neuronal excitability. Since small conductance Ca^2+^-dependent K^+^ (SK) channels modulate neuronal excitability and certain types of learning and memory, pharmacological modulation of SK channels could be used to regulate IL excitability and fear extinction. To test this, we first determined the effect of blocking SK channels with apamin on the intrinsic excitability of IL pyramidal neurons in brain slices. In whole-cell patch-clamp recordings, apamin increased the number of spikes evoked by a depolarizing current pulse, increased the firing frequency, and reduced the fast afterhyperpolarizing potential (fAHP) indicating that blockade of SK channels could be used to enhance the intrinsic excitability of IL neurons. Next, we assessed whether SK channels in IL regulate extinction of conditioned fear by infusing apamin into IL of fear conditioned rats prior to extinction training. Apamin infusion did not affect conditioned freezing at the beginning of the extinction session or within-session extinction. However, the following day, apamin-infused rats showed significantly less conditioned freezing. To further examine the importance of SK channels in IL in fear extinction, we assessed the effect of the SK channel activator DCEBIO on IL neuronal excitability and fear extinction. Activation of SK channels with DCEBIO decreased the number of evoked spikes, reduced the firing frequency, and enhanced the fAHP of IL neurons. Infusion of DCEBIO into IL prior to fear extinction impaired recall of fear extinction without affecting acquisition of extinction. Taken together, these findings suggest that SK channels are involved in regulating IL excitability and extinction-induced plasticity. Therefore, SK channels are a potential target for the development of new pharmacological treatments to facilitate extinction in patients suffering from anxiety disorders.

## Introduction

Effective retrieval of the fear extinction memory is associated with increased activation of neurons in a specific subdivision of the medial prefrontal cortex (mPFC), the infralimbic (IL) cortex (Milad and Quirk, 2002, 2012; Holmes et al., 2012). Consolidation of fear extinction memory depends on NMDA, muscarinic, and metabotropic glutamate type 5 receptor (mGluR5) activation in IL (Burgos-Robles et al., 2007; Fontanez-Nuin et al., 2011; Santini et al., 2012). After extinction, IL neurons respond more robustly to the conditioned stimulus in part due to intrinsic (Santini et al., 2008) and synaptic (Pattwell et al., 2012; Sepulveda-Orengo et al., 2013) plasticity in IL induced by mGluR5 activation (Sepulveda-Orengo et al., 2013).

NMDA, muscarinic, and mGluR5 receptors generate increases in intracellular calcium that can activate small conductance Ca^2+^-dependent K^+^ (SK) channels (Sourdet et al., 2003; Faber et al., 2005; Ngo-Anh et al., 2005; Gulledge et al., 2007) resulting in reduced neuronal excitability and afterhyperpolarization potentials (AHP; Bond et al., 2005). Secondary to their calcium-dependence and localization near NMDA receptors, SK channels shunt NMDA receptor currents and can prevent the induction of synaptic plasticity needed for memory formation (Ngo-Anh et al., 2005; Hammond et al., 2006; Stackman et al., 2008; McKay et al., 2012). In addition, the excitatory effects of mGluR5 and muscarinic receptor activation are blunted by the activation of SK channels through the release of intracellular calcium (Power and Sah, 2008; El-Hassar et al., 2011). Reducing SK channel activity enhances synaptic plasticity in hippocampal slices and facilitates hippocampal-dependent learning and memory (Stackman et al., 2002; Hammond et al., 2006). Therefore, inhibiting SK channels in IL could enhance the excitatory actions of NMDA, muscarinic and mGluR5 receptors and facilitate fear extinction-induced plasticity and extinction memory.

To test whether SK channels can modify IL neuronal excitability and fear extinction memory, we examined the effects of blocking and stimulating SK channels on the intrinsic excitability of IL pyramidal neurons using whole-cell patch-clamp recordings and on fear extinction using intra-IL infusions. In this study, we demonstrate that blocking SK channels enhances IL intrinsic excitability and long-term retention of extinction memory while stimulating SK channels has the opposite effects.

## Materials and methods

### Subjects

The procedures were approved by the Institutional Animal Care and Use Committee (IACUC) of the Ponce School of Medicine and Health Sciences in compliance with NIH guidelines for the care and use of laboratory animals. Male Sprague-Dawley rats (25–30 days postnatal) were transported from the Ponce School of Medicine and Health Sciences colony to a satellite facility where they were housed in transparent polyethylene cages inside a negative-pressure Biobubble (Colorado Clean Room, Ft. Collins, CO). Rats were maintained on a 12/12 h light/dark schedule with free access to food (standard laboratory rat chow) and water.

### Slice preparation and recordings

Naive rats (P30) were deeply anesthetized with pentobarbital (150 mg/kg), and were perfused through the heart with ice cold high sucrose solution: 252 mM sucrose, 2 mM KCl, 1.25 mM NaH_2_PO_4_, 3 mM MgSO_4_, 26 mM NaHCO_3_, 20 mM glucose and 1 mM CaCl_2_. Brains were quickly removed and placed in ice cold artificial cerebral spinal fluid (ACSF) containing 126 mM NaCl, 3 mM KCl, 1.25 mM NaH_2_PO_4_, 1 mM MgSO_4_, 26 mM NaHCO_3_, 20 mM glucose and 2 mM CaCl_2_ and bubbled with 95% O_2_ and 5% CO_2_. Coronal slices of the mPFC were cut at a thickness of 300 µm with a Vibratome 1000 Plus (Vibratome, St. Louis, MO). Slices were incubated at room temperature in ACSF for at least an hour prior to experiments. The NMDA receptor blocker MK-801 (10 µM) was added during the incubation of slices to increase neuronal survival (Schurr et al., 1995).

Slices were transferred to a submersion recording chamber and perfused at 2–3 mL/min with room temperature ACSF. Neurons were visualized with infrared video microscopy using a 40x water immersion objective on an upright E600FN microscope (Nikon Instruments, Melville, NY). Whole-cell recordings were done with glass pipettes with a resistance of 3–5 MΩ when filled with an internal solution containing KCl (20), Kgluconate (115), HEPES (10), sodium phosphocreatine (10), biocytin (10), ATP (2) and GTP (3); pH was adjusted to 7.3 with KOH (290 mOsm).

Whole-cell current-clamp recordings were obtained from the soma of mPFC pyramidal neurons located in layers II/III and V of IL. Cells were held in current-clamp mode at −60 mV and action potential discharges in response to the injection of depolarizing current pulses were recorded with a patch-clamp amplifier (MultiClamp 700A, Axon Instruments, Union City, CA). Recordings were filtered at 4 kHz, digitized at 10 kHz, and saved to computer using pCLAMP9 (Axon Instruments, Union City, CA). Membrane potentials were not corrected for the junction potential of 9 mV. The input resistance was measured from a 5 mV, 50 ms depolarizing pulse in voltage-clamp mode. To measure SK currents, IL neurons were held in voltage-clamp at a holding potential of −50 mV and an 800 ms depolarizing pulse to 0 mV was used to evoke an outward current. Tetrodotoxin (1 µM) and tetraethylammonium (1 mM) were included in the bath to block voltage-gated Na^+^ channels and some voltage-gated K^+^ channels, respectively. The internal solution for voltage-clamp recordings contained (in mM): KCl (12), Kgluconate (130), HEPES (10), sodium phosphocreatine (10), biocytin (5), ATP (2) and GTP (0.3); pH was adjusted to 7.3 with KOH and sucrose was added to adjust osmolarity to 300 mOsm.

### Morphological analysis

Biocytin (5 mM) was included in the recording solution to label the neurons for *post hoc* morphological identification of IL pyramidal neurons. At the end of the electrophysiological recordings, the slices were fixed overnight in 4% paraformaldehyde. Neurons were subsequently visualized with a standard advidin-biotin peroxidase procedure (Vectastain ABC kit, Vector Laboratories, Burlingame, CA) as previously described (Porter et al., 2001) and visualized with brightfield microscopy.

### Behavioral apparatus

Rats were fear conditioned, extinguished and tested in a chamber of 25 × 29 × 28 cm with aluminum and Plexiglas walls (Coulbourn Inst., Allentown, PA). The floor consisted of stainless steel bars that could be electrified to deliver a mild shock. A speaker was mounted on the outside wall and illumination was provided by a single overhead light. The chamber was situated inside a sound-attenuating box (Med Associates, Burlington, VT) with a ventilating fan, which produced an ambient noise level of 60 dB. The conditioned stimulus (CS) was a 4 kHz tone with duration of 30 s and an intensity of 80 dB. The unconditioned stimulus (US) was a 0.4 mA scrambled footshock, 0.5 s in duration, which co-terminated with the tone during the conditioning phase. Between sessions, floor trays and shock bars were cleaned with soapy water and the chamber walls were wiped with a damp cloth. Behavior was recorded with digital video cameras (Micro Video Products, Ontario, Canada).

### Surgery

Rats were anesthetized with ketamine and xylazine (10 ml/100 gr) and placed in a stereotaxic apparatus. After anesthesia, the skin was retracted and holes were drilled in the skull. Rats were implanted with a single 26 gauge stainless-steel guide cannula (Plastics One, Roanoke, VA) in the mPFC as described previously (Santini et al., 2004). Stereotaxic coordinates aiming towards the infralimbic cortex were 2.8 mm anterior, 1.0 mm lateral, and 4.1 mm ventral from bregma (Paxinos and Watson, 1986), with the cannula angled 11° toward the midline in the coronal plane. Rats were allowed 7 days to recover from surgery.

### Drugs and infusion procedure

Ten minutes before extinction training, apamin (10 µM, Ascent Scientific, USA) or 5,6-Dichloro-1-ethyl-1,3-dihydro-2*H*-benzimidazol-2-one (DCEBIO, 1 mM, Tocris Bioscience, USA) were infused into the mPFC. Apamin was dissolved in artificial cerebrospinal fluid and DCEBIO was dissolved in 10% DMSO. For the infusions, cannula-dummies were removed from guide cannulas and replaced with 33 gauge injectors, which were connected by polyethylene tubing (PE-20; Small Parts Inc., Miami Lakes, FL) to 5 µl syringes mounted in an infusion pump (Harvard Apparatus, Holliston, MA). Drugs were infused at a rate of 0.5 µl/min for 1 min.

### Behavioral procedure

On day 1, rats (approximately P30) received 3 tone-shock pairings (Conditioning phase). After matching for equivalent levels of freezing conditioned rats were divided into the vehicle group (Veh) and the drug group (apamin or DCEBIO). On day 2, rats were infused with vehicle or drug followed by either 8 or 15 tone-alone trials (Extinction phase). Rats infused with apamin were exposed to eight tones to induce a partial extinction, since we expected apamin to enhance extinction. Rats that received DCEBIO infusions received 15 tone trials to induce a more complete extinction, since we anticipated that DCEBIO would impair extinction. On day 3, rats received two tone-alone trials in the same chamber to test for recall of extinction (Test phase).

### Statistical analysis

The percent of time spent freezing (Blanchard and Blanchard, 1972) was used as a measure of conditioned fear. Freezing is the cessation of all movements except respiration. The total time spent freezing during the 30 s tone was scored from videotape with a digital stopwatch by observers blinded with respect to experimental group. The electrophysiological data were analyzed using Clampfit (Axon Instruments, Union City, CA). Student’s *t*-test or one-way ANOVA (STATISTICA, Statsoft, Tulsa, OK) were used to analyze the behavioral and electrophysiological data. Following a significant main effect, *post-hoc* tests were performed with Tukey honest significant difference (HSD) tests. Values are reported as the mean ± the standard error of the mean (S.E.M.).

## Results

### Blockade of SK channels increased the number of evoked spikes and burst firing in IL pyramidal neurons

**Figure 1:**
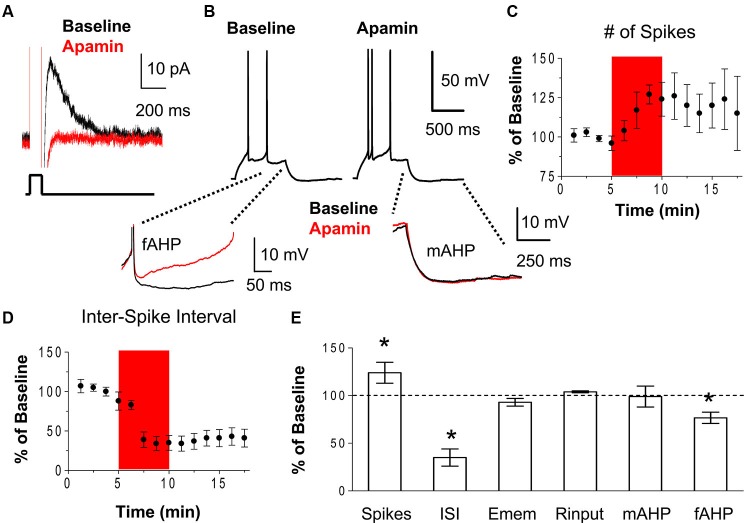
**Blockade of SK channels with apamin increases the intrinsic excitability of IL pyramidal neurons. (A)** Voltage-clamp recordings showing that bath application of the SK channel antagonist apamin (100 nM) blocked the *I*_AHP_. **(B)** Traces showing the number of spikes evoked by a current pulse during baseline and following the application of apamin. Inserts below show the overlapping fAHPs, following the second spike, and the mAHPs from the same traces. **(C–D)** Time courses showing that apamin (red area) persistently increased of the number of spikes and decreased the first ISI, consistent with enhanced bursting in IL neurons. **(E)** Summary of the effects of apamin on spike count, ISI, resting membrane potential (Emem), input resistance (Rinput), mAHP, and fAHP. *n* = 5; * *p* < 0.05.

First, we examined whether SK channels modulate the excitability of IL neurons by assessing the effect of the SK channel blocker, apamin, on the intrinsic excitability of IL pyramidal neurons using whole-cell patch-clamp recordings in coronal slices of the mPFC. Neuronal excitability was measured as the number of spikes evoked by depolarizing current steps and the first inter-spike interval (ISI). Figure 1A shows that bath perfusion of apamin (100 nM) blocks the AHP current (*I*_AHP_) in IL pyramidal neurons (0.04% of baseline, *t* = 5.28, *df* = 2, *p* = 0.03). As shown in Figures 1B–E, apamin caused a persistent increase in the number of spikes evoked by a constant depolarizing current pulse (124% of baseline, *n* = 5, *t* = 4.24, *df* = 4, *p* = 0.01) and a decrease in the first ISI (35% of baseline, *n* = 5, *t* = 5.28, *df* = 4, *p* = 0.006). These results indicate that blocking SK channels increases the intrinsic excitability and burst firing of IL pyramidal neurons. The resting membrane potential and the input resistance were not affected by apamin indicating a lack of open SK channels at the resting membrane potential and suggesting that apamin preferentially affects active neurons (Figure 1E). Consistent with a previous study (Gu et al., 2008), apamin did not reduce the medium afterhyperpolarizing potential (mAHP) measured as the peak of the AHP at the end of the depolarizing pulse (Figures 1B, E). However, apamin did reduce the fast afterhyperpolarizing potential (fAHP; 77% of baseline, *n* = 6, *t* = 3.98, *df* = 5, *p* = 0.01) which was measured as the peak AHP between the second and third spike subtracted from the threshold potential for spike initiation (Figures 1B, E).

### Blockade of SK channels facilitated extinction recall

**Figure 2:**
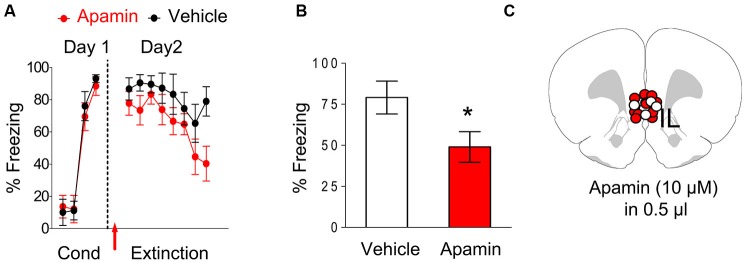
**Infusion of apamin into IL prior to extinction training facilitated extinction recall. (A)** Freezing to the tone during fear conditioning and extinction for vehicle-infused rats (*n* = 7) and rats infused with apamin (*n* = 11) into IL. Arrow indicates the time of the infusion. **(B)** Average freezing to two test tones on day 3. Apamin-infused rats showed reduced fear expression on day 3, consistent with enhanced recall of extinction memory. **(C)** White dots vehicle group (VEH) and red dots (apamin) represent the cannula placements for infusions.

After showing that blocking SK channels enhances IL neuronal excitability in slices, we tested whether blocking SK channels in IL could facilitate extinction of conditioned fear. Rats were fear conditioned with three tone-shock pairings on day 1 (Figure 2A). Both groups showed similar levels of freezing to the last tone of conditioning (apamin 88%, vehicle 93%, *t* = 0.59, *df* = 16, *p* = 0.56). The next day, rats received intra-IL infusions of apamin (10 µM; *n* = 11) or vehicle (*n* = 7) prior to extinction. Both groups showed similar levels of freezing to the first tone of extinction (apamin 78%, saline 87%, *t* = 0.82, *df* = 16, *p* = 0.42) indicating that apamin did not affect recall of the conditioned fear. Both groups also acquired similar levels of extinction. A repeated-measures ANOVA across the extinction trials of day 2 showed no trial by group interaction (*F*_(7, 112)_ = 0.66, *p* = 0.70) indicating that apamin did not significantly affect acquisition of extinction. However, on day 3, the rats that received the intra-IL infusion of apamin showed reduced fear expression (mean freezing day 3, apamin 49%, saline 79%; *t* = 2.12, *df* = 16, *p* = 0.05), indicating that SK channel blockade in IL facilitates the recall of extinction memory (Figures 2B–C).

### Stimulation of SK channels reduced the number of evoked spikes and burst firing in IL pyramidal neurons

**Figure 3:**
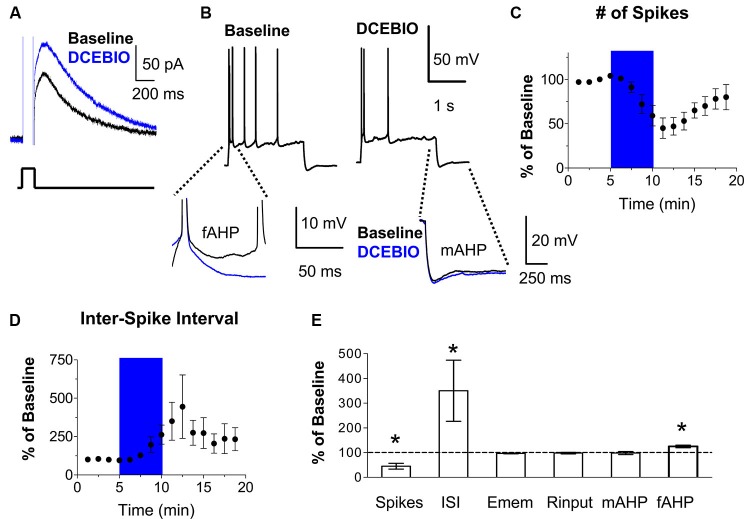
**Stimulation of SK channels with DCEBIO reduced the intrinsic excitability of IL pyramidal neurons. (A)** Voltage-clamp recordings showing that bath application of the SK channel agonist DCEBIO (30 µM) enhanced the *I*_AHP_. **(B)** Traces showing the number of spikes evoked by a current pulse during baseline and perfusion of DCEBIO. Inserts below show the overlapping fAHPs, following the second spike, and the mAHPs from the same traces. **(C–D)** Time courses showing that application of DCEBIO (blue area) decreased the number of evoked spikes and increased the first ISI, consistent with reduced bursting in IL. **(E)** Summary of the effects of DCEBIO on spike count, ISI, Emem, Rinput, mAHP, and fAHP. *n* = 5; * *p* < 0.05.

Next, we tested whether stimulation of SK channels with the SK channel activator DCEBIO (Pedarzani et al., 2005) depresses the intrinsic excitability of IL neurons. Consistent with previous findings (Pedarzani et al., 2005), DCEBIO enhanced the *I*_AHP_ in IL neurons (Figure 3A). Recording action potentials in response to an injected current pulse showed that DCEBIO (30 µM) decreased the number of evoked spikes in IL pyramidal neurons (45% of baseline, *n* = 5, *t* = 5.67, *df* = 4, *p* = 0.005; Figures 3B–C). Figure 3D shows that DCEBIO also increased the first ISI (262% of baseline, *n* = 5, *t* = 2.77, *df* = 4, *p* = 0.05) indicating that SK channels reduce bursting in IL neurons. DCEBIO also increased the fAHP (125% of baseline, *n* = 5, *t* = 5.35, *df* = 4, *p* = 0.006). As with apamin, DCEBIO did not affect the resting membrane potential, input resistance, or mAHP (Figures 3B, E).

### Stimulation of SK channels in IL impaired recall of fear extinction

**Figure 4:**
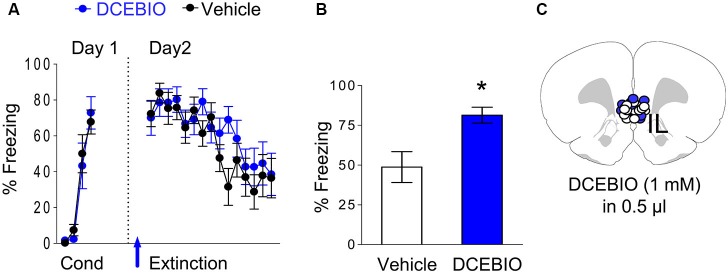
**Infusion of DCEBIO into IL prior to extinction impaired extinction recall. (A)** Freezing to the tone for vehicle-infused rats (*n* = 12) and rats infused with DCEBIO (*n* = 12) into IL. Arrow indicates the time of the infusions. **(B)** Average freezing to two test tones on day 3. DCEBIO-infused rats showed enhanced fear expression on day 3, consistent with impaired recall of extinction memory. **(C)** White dots (VEH) and blue dots (DCEBIO) represent the cannula placements for infusions.

Since blocking SK channels in IL enhanced recall of extinction memory, we hypothesized that stimulation of these channels would impair extinction recall. To test this, we examined the effect of infusing DCEBIO (1 mM) into IL on fear extinction. Rats received fear conditioning consisting of three tone-shock pairings, and 24 h later they were exposed to 15 tone-alone extinction trials 10 min after an intra-IL infusion of DCEBIO (*n* = 12) or vehicle (*n* = 12). As shown in Figure 4A, both groups were matched for similar levels of freezing to the last conditioning tone (DCEBIO 72% freezing, vehicle 68% freezing, *t* = 0.46, *df* = 22, *p* = 0.65). The next day, both groups showed similar levels of freezing to the first tone of extinction (DCEBIO 70%, saline 72%, *t* = 0.18, *df* = 22, *p* = 0.86) indicating that DCEBIO did not affect recall of the conditioned fear. Infusion of DCEIO also did not affect acquisition of extinction. A repeated-measures ANOVA across the extinction trials of day 2 found no trial by group interaction (*F*_(14, 308)_ = 1.04, *p* = 0.41). However, the next day DCEBIO-infused rats showed enhanced fear expression (DCEBIO 81% vs. vehicle 49% freezing; *t* = 2.99, *df* = 22, *p* = 0.007), indicating that activation of SK channels in IL impaired recall of extinction memory (Figures 4B–C).

## Discussion

Despite an increased understanding of the role of SK channels in the process of memory formation (Kuiper et al., 2012), no previous study has examined whether SK channels are involved in extinction of conditioned fear. In this study, we examined the effect of SK channel blockade and activation on IL intrinsic excitability and recall of extinction memory. Our findings show that blocking SK channels with apamin enhances the intrinsic excitability of IL pyramidal neurons. In addition, rats infused with apamin directly into IL prior fear extinction training showed facilitated extinction recall. Conversely, stimulation of SK channels with DCEBIO depressed the intrinsic excitability of IL pyramidal neurons and infusion of DCEBIO into IL prior to fear extinction impaired extinction recall. Together our findings suggest that SK channels in IL modulate the neuronal plasticity required to form a long-term extinction memory.

SK channels could modulate extinction by reducing NMDA signaling in IL. Recall of fear extinction is impaired by blocking NMDA receptors in IL during extinction training (Burgos-Robles et al., 2007) indicating that the formation of extinction memory requires NMDA receptor-dependent plasticity in IL. Calcium influx through NMDA receptors activates SK channels which reduce the duration of NMDA-mediated depolarization and calcium influx into dendritic spines (Ngo-Anh et al., 2005) and can, thereby, impair synaptic integration and NMDA receptor-dependent synaptic plasticity (Stackman et al., 2002). Therefore, apamin could enhance consolidation of the extinction memory by relieving this brake on NMDA-dependent synaptic plasticity. In support of this hypothesis, apamin enhances NMDA currents in pyramidal neurons in the mPFC (Faber, 2010).

Blocking SK channels could also enhance fear extinction memory by increasing signaling via mGluR5 and muscarinic receptors in IL which also contribute to the consolidation of fear extinction memory (Fontanez-Nuin et al., 2011; Santini et al., 2012; Sepulveda-Orengo et al., 2013). Both mGluR5 and muscarinic receptors induce the production of inositol 1, 4, 5-trisphosphate which releases intracellular calcium to activate SK channels (Gulledge and Kawaguchi, 2007; Hagenston et al., 2008; El-Hassar et al., 2011; Clements et al., 2013). The activation of SK channels on dendritic spines suppresses synaptic currents and calcium signaling to reduce synaptic plasticity (Giessel and Sabatini, 2010). Therefore, apamin could facilitate extinction memory by enhancing mGluR5 or muscarinic receptor-mediated plasticity in IL.

Apamin enhanced spike firing without affecting the resting membrane potential suggesting that apamin would primarily affect actively firing neurons such as those responding to the tone CS during extinction training (Milad and Quirk, 2002; Holmes et al., 2012). The increased neuronal firing would allow for stronger Hebbian plasticity (Cooper, 2005) and increase the probability that these neurons would form part of the circuit consolidating the extinction memory (Zhou et al., 2009). In support of this possibility, apamin enhanced recall of fear extinction without affecting fear expression or extinction learning suggesting that apamin enhanced extinction recall primarily by facilitating consolidation of extinction. Consistent with our findings, systemic activation of SK channels does not affect fear expression suggesting that SK channels in other structures also do not modulate fear expression (Atchley et al., 2012). In contrast to the effects of apamin, blocking M-type K+ channels depolarized IL neurons and reduced fear expression at the beginning of extinction training (Santini and Porter, 2010) suggesting that different K+ channels in IL regulate fear expression and extinction plasticity. However this relationship may be altered by stress, since systemic activation of SK channels reduces conditioned fear expression after repeated stress (Atchley et al., 2012).

Our findings expand the growing literature showing that SK channels regulate various types of learning and memory (Kuiper et al., 2012). Blocking SK channels enhances hippocampal-dependent spatial, object recognition, and contextual fear memory (Stackman et al., 2002; Vick et al., 2010) and prefrontal-dependent spatial working memory (Brennan et al., 2008). Furthermore, overexpression or pharmacological activation of SK channels impairs hippocampal- and amygdala-dependent learning and memory (Blank et al., 2003; Hammond et al., 2006; McKay et al., 2012). Thus SK channels appear to be key regulators of memory consolidation.

To overcome the extinction-deficits seen in post-traumatic stress disorder (PTSD) patients (Milad et al., 2009), there is considerable interest in developing compounds that enhance NMDA receptor signaling to facilitate extinction memory (Davis, 2011). One compound designed to enhance NMDA receptor signaling, D-cycloserine (Walker et al., 2002), has shown promise in improving PTSD symptoms (de Kleine et al., 2012; Difede et al., 2014). Given their potential for potentiating NMDA receptor activity, SK channel inhibitors present a new alternative that could be used in combination with D-cycloserine to increase the effectiveness of behavioral therapies for patients suffering from anxiety disorders such as PTSD. Since blocking SK channels systemically could increase fear by activating fear promoting structures such as the prelimbic cortex and amygdala (Peters et al., 2009; Mahan and Ressler, 2012; Marek et al., 2013), future studies will need to determine the net affect of systemic SK channel antagonists on fear extinction.

## Conflict of interest statement

The authors declare that the research was conducted in the absence of any commercial or financial relationships that could be construed as a potential conflict of interest.
